# Maternal Allergic Asthma Induces Prenatal Neuroinflammation

**DOI:** 10.3390/brainsci12081041

**Published:** 2022-08-05

**Authors:** Juan M. Tamayo, Destanie Rose, Jamie S. Church, Jared J. Schwartzer, Paul Ashwood

**Affiliations:** 1 Department of Medical Microbiology and Immunology, University of California, Davis, CA 95817, USA; 2 The M.I.N.D. Institute, University of California Davis, Sacramento, CA 95817, USA; 3 Program in Neuroscience and Behavior, Department of Psychology and Education, Mount Holyoke College, 50 College Street, South Hadley, MA 01075, USA

**Keywords:** mouse model, neurodevelopment, cytokines, autism spectrum disorder, schizophrenia, ADHD, pregnancy, neuroinflammation, asthma, allergy, fetal brain, placenta

## Abstract

Autism spectrum disorder (ASD) is a class of neurodevelopmental disorders characterized by impaired social interactions and communication skills and repetitive or stereotyped behaviors. Rates of ASD diagnosis continue to rise, with current estimates at 1 in 44 children in the US (Maenner 2021). Epidemiological studies have suggested a link between maternal allergic asthma and an increased likelihood of having a child diagnosed with ASD. However, a lack of robust laboratory models prevents mechanistic research from being carried out. We developed a novel mouse model of maternal asthma-allergy (MAA) and previously reported that offspring from these mothers exhibit behavioral deficits compared to controls. In addition, it was shown that epigenetic regulation of gene expression in microglia was altered in these offspring, including several autism candidate genes. To further elucidate if there is neuroinflammation in the fetus following MAA, we investigated how allergic asthma impacts the maternal environment and inflammatory markers in the placenta and fetal brain during gestation. Female C57Bl/6 mice were primed with ovalbumin (OVA) prior to allergic asthma induction during pregnancy by administering aerosolized ovalbumin or PBS control to pregnant dams at gestational days (GD)9.5, 12.5, and 17.5. Four hours after the final induction, placenta and fetal brains were collected and measured for changes in cytokines using a Luminex bead-based multiplex assay. Placental MAA tissue showed a decrease in interleukin (IL)-17 in male and female offspring. There was a sex-dependent decrease in female monocyte chemoattractant protein 1 (MCP-1). In male placentas, IL-4, C–X–C motif chemokine 10 (CXCL10)—also known as interferon γ-induced protein 10 kDa (IP-10)—and chemokine (C-C motif) ligand 5 (RANTES) were decreased. In fetal brains, elevated inflammatory cytokines were found in MAA offspring when compared to controls. Specifically, interferon-gamma (IFN-γ), granulocyte-macrophage colony-stimulating factor (GM-CSF), interleukin 1α (IL-1α), IL-6, and tumor necrosis factor α (TNFα) were elevated in both males and females. In contrast, a decrease in the cytokine IL-9 was also observed. There were slight sex differences after OVA exposures. Male fetal brains showed elevated levels of macrophage inflammatory protein-2 (MIP-2), whereas female brains showed increased keratinocytes-derived chemokine (KC). In addition, IL-1𝛽 and IP-10 in male fetal brains were decreased. Together, these data indicate that repeated exposure to allergic asthma during pregnancy alters cytokine expression in the fetal environment in a sex-dependent way, resulting in homeostatic and neuroinflammatory alterations in the fetal brain.

## 1. Introduction

Autism spectrum disorder (ASD) is a neurodevelopmental disorder characterized by impaired social interactions and communications skills and repetitive or stereotyped behaviors. Rates of ASD diagnosis continue to rise, with current estimates at 1 in 44 children in the US [1]. ASD appears to impact males more frequently than females, with a 4:1 ratio, respectively [2,3]. While the etiology of ASD is unknown, research has identified both genetic and environmental contributing factors. Many of these suspected contributors are shared among other neuropsychiatric disorders, such as attention-deficit/hyperactive disorder (ADHD) and schizophrenia. Some reports put the genetic risk for ASD at over 50%, although the presence of specific genes associated with ASD does not guarantee that an individual will later be diagnosed with ASD. That is, many of the risk genes associated with ASD represent common genetic variants in the population that carry low risk [4]. This moderate contribution of genetics to the development of ASD underscores the importance of various environmental factors, particularly during fetal development, that likely contribute to increased risk and severity of neurodevelopmental disorders (NDD).

Epidemiology studies have begun to uncover some of the underlying environmental etiologies, with a focus on the immune system. Environmental factors, such as maternal exposure to stress, obesity, pollution, infection, and allergy/asthma during pregnancy, can increase the likelihood of having a child later diagnosed with ASD. Animal models of these environmental risk factors suggest that there are behavioral and transcriptional changes in offspring as a result of cytokine signaling mediated by the placenta [5]. A common theme among these models is the activation of the maternal immune system. For example, one of the most widely studied environmental factors contributing to an increased likelihood of NDD in mice is the activation of the maternal immune system during pregnancy using bacterial or viral mimics [5]. Although infectious agents have been widely studied, recent meta-analyses of viral infections during pregnancy as a risk for humans with ASD was not supported [6]. Rates of asthma, however, are currently on the rise, and multiple studies have shown that mothers with asthma are more likely to have asthma exacerbations during pregnancy and subsequently a child with an ASD diagnosis [7,8,9,10,11,12,13,14,15,16]. Moreover, increased asthma severity was linked to increased ASD risk, independent of medication usage [11]. In early reports, Croen et al. showed that childhood ASD was associated with mothers’ asthma diagnoses in the first and second trimesters [10]. In addition, maternal asthma has also been linked with other NDD, such as ADHD [17,18,19]. Although these studies suggest a link between maternal asthma and later diagnosis of ASD, there are very few studies that have directly investigated the impact that asthma has on the developing fetal brain and its environment during gestation.

Recently, our laboratory developed a model of maternal allergic asthma (MAA) in which offspring mimic behavioral outcomes relevant to the core features of ASD, including decreased social interaction and repetitive-like behaviors [20,21,22]. Within this model, MAA exposure results in changes in gene expression in microglia of adult offspring, highlighting the lasting epigenetic impact of MAA on the offspring’s neuroimmune environment [23]. These behavioral and neuroimmune changes in offspring occur in response to allergic asthma-associated elevations in inflammatory cytokines in the dams—namely, IL-4, IL-5, IL-6, and IL-17 [20]—and closely mirror observations reported in clinical settings. Specifically, in a case control study by Goines et al., elevated levels of IL-4 and IL-5 during mid-pregnancy were shown to be associated with mothers who had children with autism [24]. In further studies, high levels of IL-4 were detected in the amniotic fluid and newborn blood spots of children who were later diagnosed with ASD [13,25]. These observations in humans demonstrate an important role for the maternal inflammatory environment in shaping risk for ASD in the offspring and support the validity of our mouse model of MAA in identifying potential mechanistic links.

Maternal systemic inflammation can alter the fetal environment and have both short- and long-term consequences on the developing brain [26]. For example, Zaretsky et al. reported that IL-6 can cross the human placenta in both the maternal–fetal and fetal–maternal directions [27]. This phenomenon has also been demonstrated in rat models, which showed that IL-6 administration during mid- or late pregnancy can transfer across the placental barrier to reach the fetus [28]. In addition, mouse fetal brain cytokine expression of IL-1β, IL-6, IL-17, IL-13, MCP-1, and monocyte inflammatory protein-1 alpha (MIP-1α) are altered hours after an inflammatory viral insult to the dam [29,30,31]. While these studies show a link between viral/bacterial maternal infection and changes in the fetal brain, it has yet to be established in a model of MAA.

Given the links in humans, and the observed behavioral and neuroimmunological impacts of MAA in mice, it can be reasoned that MAA-induced changes in the offspring brain may begin at the fetal stages of development in response to elevated allergic-asthma associated cytokines. In these studies, we hypothesized that MAA results in sex-dependent changes in the fetal brain and placenta cytokine expression following allergic asthma episodes. To test this hypothesis, our laboratory collected placental and fetal brain tissue following MAA exposure to investigate whether the inflammatory environment in these tissues is altered in response to this maternal insult.

## 2. Methods

### 2.1. Animals

Male and female C57BL/6J mice generated from breeding pairs purchased from Jackson Laboratory (Bar Harbor, MA, USA) were bred and maintained at Mount Holyoke College, South Hadley, MA. Mice were raised on a 12 h light/dark cycle (lights on at 0800 h) and group-housed in individually ventilated cages with same-sex littermates until breeding at 8 weeks of age. Cages were maintained in a temperature-controlled (23 °C) vivarium with food and water provided *ad libitum*. All procedures were performed with approval by the Mount Holyoke College Institutional Animal Care and Use Committee and in accordance with the guidelines provided by the National Institutes of Health Guide for the Care and Use of Laboratory Animals.

### 2.2. Maternal Allergic Asthma Induction

Allergic asthma inductions were carried out using procedures previously described [20,21]. Briefly, sexually naïve female mice were sensitized with 10 µg ovalbumin (OVA, Sigma, St Louis, MO, USA) and 1 mg (Al)OH_3_ (Invitrogen, San Diego, CA, USA) dissolved in 200 µL of phosphate-buffered saline (PBS) injected intraperitoneally at 6 and 7 weeks of age. Beginning at 8 weeks of age, females were mated overnight, the presence of a seminal plug was checked daily—noted as gestational day (GD)0.5—and female mice were single-housed. Pregnant mice were randomly assigned to receive either an aerosolized solution of 1% (wt/vl) OVA in PBS (n = 10) or PBS alone (n = 8) for three 45 min induction sessions throughout gestation. Specifically, these induction sessions occurred at gestational days 9.5, 12.5, and 17.5 to correspond to early, middle, and late gestation, as previously described [20,21]. 

### 2.3. Serum, Placenta, and Fetal Brain Collection

Four hours after the final induction, mice were anesthetized with isoflurane (2–4% inhalation) and 500 µL of whole blood was collected from dams via cardiac puncture. Blood was allowed to clot at room temperature for 30 min and centrifuged at 10,000× *g* for 10 min at 4 °C, then serum was collected and stored at −80 °C. In addition, each placenta and fetal brain was extracted, flash frozen in liquid nitrogen, and individually stored at −80 °C until further processing. To determine the sex of the specimens, placenta and brain were genotyped for the presence or absence of SRY using polymerase chain reaction (PCR). A total of 74 brains and placenta were extracted from the 18 experimental litters, resulting in the following groups: PBS male (n = 15), PBS female (n = 18), MAA male (n = 23), and MAA female (n = 18).

### 2.4. Placenta and Fetal Brain Tissue Processing

Placenta and brain tissue were lysed using cell lysis buffer (Cell Signaling Technologies, Danvers, MA, USA) containing protease and phosphatase inhibitors. The tissue was incubated in lysis buffer with agitation for 20 min on ice followed by sonication for 30 s. Cell lysate was then vortexed at top speed for 30 s and centrifuged at 20,000× *g* for 10 min at 4 °C. Protein concentrations were measured using a Bio-Rad Benchmark Plus Spectrophotometer system and all samples were standardized to 70 µg/mL for subsequent immunoassays.

### 2.5. Multiplex Bead-Based Cytokine Analysis

Analysis of serum cytokines was performed using a multiplex mouse 25-plex bead immunoassay (Milliplex Mouse Cytokine/Chemokine Magnetic Bead Panel #MCYTMAG70PMX25BK). The following cytokines were quantified: G-CSF, GM-CSF, IFN-γ, IL-1α, IL-1β, IL-2, IL-4, IL-5, IL-6, IL-7, IL-9, IL-10, IL-12 (p40), IL-12 (p70), IL-13, IL-15, IL-17, IP-10, KC, MCP-1, MIP-1α, MIP-1β, MIP-2, RANTES, and TNF-α. Standards and reagents were all prepared according to the manufacturers’ recommendations. Each serum, brain, and placenta sample was diluted to a standardized concentration and run in duplicate. Twenty-five microliters of sample, standards, or blanks were loaded into a 96-well plate with appropriate amounts of assay buffer and matrix solution. The plate was then incubated overnight with antibody-coupled magnetic beads. The following day, after a series of washes, the plate was incubated with a biotinylated detection antibody on a shaker for 1 h. Streptavidin-phycoerythrin was added and incubated while shaking continued for 30 min. Washes were undertaken using a Bio-Plex handheld magnet (Bio-Rad Laboratories, Hercules, CA, USA). After the final wash, the plate was analyzed using a Bio-Rad Bio-Plex 200 plate reader (Bio-Rad Laboratories, Hercules, CA, USA) and analyzed using Bio-Plex Manager software (Bio-Rad Laboratories, Hercules, CA, USA). The following were the minimal amounts of detectable cytokine concentrations: G-CSF: 1.7 pg/mL; GM-CSF: 10.9 pg/mL; IFNγ: 1.1 pg/mL; IL-1α: 10.3 pg/mL; IL-1β: 5.4 pg/mL; IL-2: 1.0 pg/mL; IL-4: 0.4 pg/mL; IL-5: 1.0 pg/mL; IL-6: 1.1 pg/mL; IL-7: 1.4 pg/mL; IL-9: 17.3 pg/mL; IL-10: 2.0 pg/mL; IL-12 (p40): 3.9 pg/mL; IL-12 (p70): 4.8 pg/mL; IL-13: 7.8 pg/mL; IL-15: 7.4 pg/mL; IL-17: 0.5 pg/mL; IP-10: 0.8 pg/mL; KC: 2.3 pg/mL; MCP-1: 6.7 pg/mL; MIP-1α: 7.7 pg/mL; MIP-1β: 11.9 pg/mL; MIP-2: 30.6 pg/mL; RANTES: 2.7 pg/mL; TNF-α: 2.3 pg/mL. Sample concentrations that fell below minimal detection value were given a proxy value of half the limit of detection for statistical comparisons.

### 2.6. Statistical Analysis

Data were analyzed using GraphPad Prism Version 9.3.1 (GraphPad Software, San Diego, CA, USA) and RStudio version 1.4.1106 (2021) (RStudio, PBC, Boston, MA, USA) using the “nlme” package. Maternal serum cytokine concentrations between MAA and PBS dams were assessed using non-parametric Mann–Whitney U analyses. Similarly, placenta data was in lower concentrations and did not meet the assumptions of normality; therefore, placenta cytokines were pooled within the litter and differences between OVA and PBS were assessed using non-parametric Mann–Whitney U analyses to increase signal detection. To control for pseudoreplications and litter-to-litter variations, offspring brain cytokine measures were evaluated separately for male and female mice using multilevel modeling to control for type I error [32,33], with offspring as the level 1 measure nested in dams as the level 2 two variable. Treatment (MAA or PBS) was set as the fixed effect and litter as the random effect. Model estimates with a *p*-value less than 0.05 were considered significant.

## 3. Results

### 3.1. Maternal Serum Cytokines

**Figure 1:**
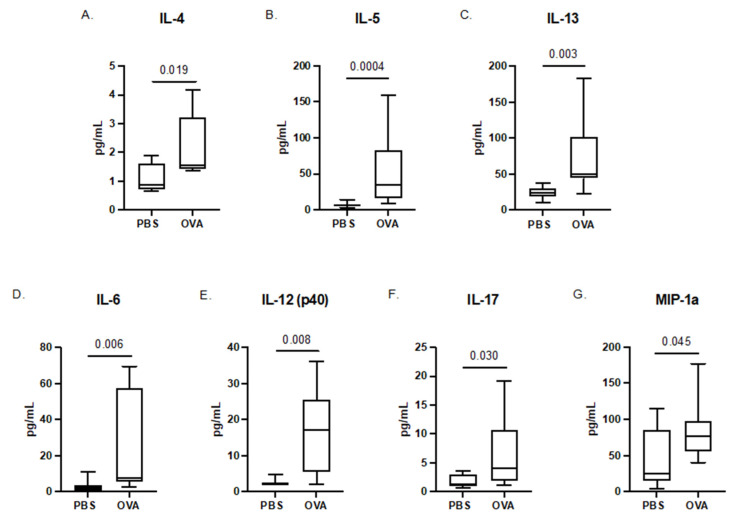
Cytokine concentrations in maternal sera of dams exposed to PBS and OVA taken at GD17.5. Serum was collected prior to fetal offspring and placenta collections. Cytokine levels were assessed using a multiplex bead-based immunoassay. (**A**) IL-4, (**B**) IL-5, (**C**) IL-13, (**D**) IL-6, (**E**) IL-12 (p40), (**F**) IL-17, and (**G**) MIP-1α are represented as pg/mL after being normalized to total protein content. Statistical significance determined by Mann–Whitney U test. PBS dams n = 8, OVA dams n = 10.

Following the final induction at GD17.5, the maternal sera were collected from dams of both treatment groups. Maternal serum in the MAA dams showed elevated levels of the T-helper type 2 allergic asthma-associated cytokines, including IL-4 (*p* = 0.019), IL-5 (*p* = 0.0004), and IL-13 (*p* = 0.003) [34,35,36], confirming an allergic asthma response. Moreover, MAA dams had elevated levels of IL-6 (*p* = 0.006), IL-12 (*p* = 0.008), IL-17 (*p* = 0.030), and MIP-1α (*p* = 0.045) compared with PBS-treated control dams (Figure 1). While there were also trends for increased levels of GM-CSF and IL-10, these increases did not reach statistical significance.

### 3.2. Offspring Placenta Cytokines

**Figure 2:**
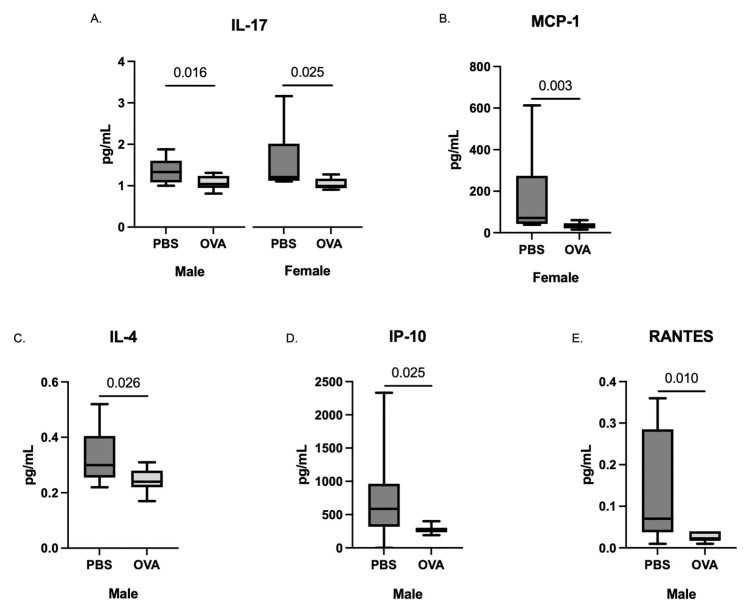
Cytokine concentrations in the placenta taken from dams exposed to PBS and OVA at GD 17.5 after final allergic asthma challenge. Cytokine levels were assessed using a multiplex bead-based immunoassay. The concentrations of (**A**) IL-17, (**B**) MCP-1, (**C**) IL-4, (**D**) IP-10, and (**E**) RANTES are represented as pg/mL after being normalized to total protein content. Statistical significance was determined by Mann–Whitney U test.

**Figure 3:**
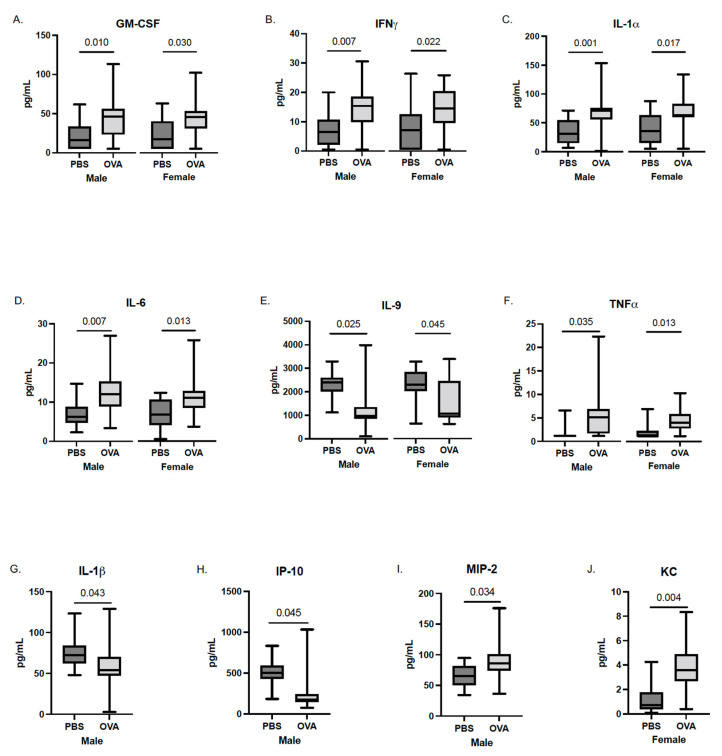
Cytokine concentrations in the fetal brain (whole-brain homogenates) taken at GD17.5 following final maternal asthma challenge. Concentrations of cytokines from male and female offspring of OVA- and PBS-exposed dams were assessed using a multiplex bead-based immunoassay. The concentrations of (**A**) GM-CSF, (**B**) IFN𝛾, (**C**) IL-1𝛼, (**D**) IL-6, (**E**) IL-9, (**F**) TNF𝛼, (**G**) IL-1𝛽, (**H**) IP-10, (**I**) MIP-2, and (**J**) KC are represented as pg/mL after being normalized to total protein content. Statistical significance determined by multilevel mixed-effects modeling.

Placentas were collected following the final induction and cytokine analysis was performed on placenta homogenates. Given that placenta data showed lower concentrations and did not meet the assumptions of normality, placenta cytokines were pooled within the litter and differences between OVA and PBS were assessed using non-parametric Mann–Whitney U analysis to increase signal detection. Placental levels of IL-17 were decreased in MAA offspring placentas for both females (*p* = 0.025) and males (*p* = 0.016) (Figure 2 and Figure 3, respectively) compared with PBS controls. In addition, in placentas from female offspring, we observed a decrease in MCP-1 (*p* = 0.003), which was not found in male counterparts. Conversely, in male placentas only, there were significant decreases in IL-4 (*p* = 0.026), IP-10 (*p* = 0.025), and RANTES (*p* = 0.010).

### 3.3. Whole-Brain Fetal Cytokines

Following the removal of the fetus from the placenta, whole fetal brains were collected, homogenized, and analyzed for cytokine concentration. Multilevel mixed-effects modeling revealed elevated levels of several cytokines in both sexes of MAA offspring, most of which are generally considered inflammatory in nature; namely, GM-CSF, IFN𝛾, IL-1𝛼, IL-6, and TNF𝛼. Specifically, MAA exposure resulted in an average 21–22 pg/mL increase in GM-CSF concentrations in both male and female (Figure 3) offspring brains compared to sex-matched offspring of PBS dams (male, b = 22.32, CI: 6.66–37.98, t(16) = 2.94, *p* = 0.010; female, b = 21.36, CI: 2.88–39.84, t(16) = 2.38, *p* = 0.030). Similar increases were also observed in both male and female offspring for IFN𝛾 (male, b = 7.07, CI: 2.35–11.79, t(16) = 3.09, *p* = 0.007; female, b = 6.48, CI: 1.24–11.72, t(16) = 2.55, *p* = 0.22), and IL-1𝛼 (male, b = 35.19, CI: 16.94–53.44, t(16) = 3.98, *p* = 0.001; female, b = 28.89, CI: 6.49–51.29, t(16) = 2.66, *p* = 0.017). Moreover, both male and female offspring from MAA dams expressed higher levels of IL-6 (male, b = 5.11, CI: 1.67–8.54, t(16) = 3.07, *p* = 0.007; female, b = 4.03, CI: 1.05–7.00, t(16) = 2.79, *p* = 0.013) and TNF𝛼 (male, b = 3.41, CI: 0.35–6.46, t(16) = 2.30, *p* = 0.035; female, b = 2.52, CI: 0.66–4.38, t(16) = 2.79, *p* = 0.013) compared to sex-matched PBS controls. A decrease in IL-9 was also associated with both male and female offspring from MAA dams compared to controls (male, b = −887.35, CI: −1626.55–−148.16, t(16) = −2.48, *p* = 0.025; female, b = −784.03, CI: −1527.74–−40.31, t(16) = −2.17, *p* = 0.045).

In addition to the broad increases in inflammatory cytokines in both sexes, there were additional sex-specific changes in several cytokines in response to MAA. In males (Figure 3) but not females, there was a significant decrease in IL-1𝛽 (b = −18.80, Cl: −36.50–−1.11, t(16) = −2.19, *p* = 0.043) and IP-10 (b = −215.76, Cl: −420.39–−11.13, t(16) = −2.18, *p* = 0.045) and a significant increase in MIP-2 (b = 21.28, Cl: 2.42–41.23, t(16) = 2.32, *p* = 0.034) compared to sex-matched PBS offspring. In addition, in MAA male offspring, an increase in G-CSF (b = 4.76, Cl: 0.04–9.49, t(16) = 2.08, *p* = 0.054) compared to PBS offspring was also observed, but it did not reach statistical significance (*p* < 0.05). Conversely, in females, MAA-exposed offspring showed an increase in KC levels compared to female offspring of PBS dams (b = 2.35, Cl: 0.90–3.79, t(16) = 3.35, *p* = 0.004). Similarly, increases in cytokines G-CSF, IL-2, MIP-2, IP-10, and MIP-1β were observed in female MAA offspring compared to PBS controls, but these did not reach statistical significance (*p* < 0.09) (data not shown).

## 4. Discussion

The fetal environment plays a vital role in offspring neurodevelopment and behavior, and the maternal response to allergies and asthma represents an increasingly common environmental factor that can impact fetal development. We hypothesized that allergic asthma exposure during pregnancy would result in sex-specific neuroinflammation in the brains of developing offspring in utero, based on previous studies on maternal asthma and ASD [16]. As predicted, the analysis of the maternal serum following MAA challenge confirmed systemic inflammation consistent with the allergic-asthma immune phenotype. These elevations in maternal cytokines were met with concomitant elevations in neuroinflammatory signals in the fetal brain. There were common cytokines elevated in fetal brains in both males and females but, additionally, several cytokines were sex-specific and may correspond to differences in behavior response seen in MAA models [20,21] and, also, in humans [16]. Interestingly, increases in fetal brain cytokines were paralleled by decreases in inflammatory cytokines in the placental tissue analysis. Together, our findings support the notion that maternal inflammation in response to MAA impacts the fetal neuroimmune environment during gestation.

In humans, an allergic asthma response is often associated with a systemic increase of IL-4 and IL-5, both of which have been linked to an increased likelihood of birthing a child later diagnosed with neurodevelopmental disorders when higher levels occur during pregnancy [24,37]. In case-control studies, Goines et al. also demonstrated that elevations in IL-5 and IL-4 together during gestation were associated with a 50% increased risk of ASD [24]. Here, we showed that our model of MAA recapitulates the exposure to cytokines that are hallmarks of the allergic asthma response, including increased levels of IL-4, IL-6, IL-5, IL-13, and IL-17. This global response may translate more closely to the human exposure than single cytokine exposures in isolation. These combined cytokine signals may be the central mechanistic factors that link MAA to the behavioral deficits—and shape brain chemistry and neurocircuitry—observed in our previous studies of this model [20,22,24].

The placenta plays the role of a multipurpose organ for the fetus, acting as the lungs, gut, kidneys, and liver [38]. In addition, the placenta acts as a communicator between the fetus and mother, relaying important environmental signals to prime the fetus to adapt to future postpartum insults [39]. For example, extravillous trophoblast cells can interact with the maternal immune system by migrating to the uterine wall from the placenta [35]. Acting in the converse direction, from mother to fetus, some cytokines, such as IL-6, which were elevated in our MAA model can cross the placental barrier [28] and directly impact fetal development. A previous report by Hsiao et al. shows that IL-6 levels remain elevated in the placentas of maternal immune-activated (MIA) dams compared to saline controls 24 h following immune induction with poly(I:C) [40]. Although we report here an elevated IL-6 response in maternal serum, the placental tissue did not mirror the findings by Hsiao et al. In fact, of the cytokines measured in our study, we did not observe significant elevations in any of the inflammatory markers measured. It is unclear yet why our findings do not mirror common changes shown in poly(I:C) models of MIA, though these differences may represent a distinct mechanistic pathway unique to allergic asthma inflammation compared to other viral/bacterial models of maternal inflammation. Moreover, our measures of placental and brain cytokine makers were taken 4 h after the final MAA induction, representing a single cross-sectional timepoint during the inflammatory cascade. Given that the cellular and molecular responses to allergic asthma change dynamically across several hours of exposures, there are likely other placental changes occurring earlier and/or later in the inflammatory time-course that were not captured in this single cross-sectional window. Future longitudinal measures are needed to capture a more complete timeline of placental changes.

Our model demonstrates that MAA induces a change in the inflammatory profile of offspring brains at GD17.5. Among these changes, increases in GM-CSF, INFγ, IL-1α, IL-6, and TNF𝛼 were found. These increases were seen in both male and female fetal brains. Postmortem tissue studies have found elevated TNF𝛼, IL-6, IFNγ, and GM-CSF in brains from ASD individuals compared to their typically developing counterparts [41]. Although our fetal brain analyses also show elevations in these cytokines, we have yet to perform this analysis on whole brains from adult mice. However, we recently showed elevated IFNγ in the hypothalamus of adult animals when exposed to MAA in early fetal development [22]. Notably, IL-6, which is normally expressed at low levels in the brain, plays a role in neurogenesis, cell growth, and myelination or demyelination. Moreover, IL-6 has been linked to altered neuronal cell adhesion, migration, and synaptic formation [42,43]. TNF𝛼 has also been shown to have an impact on neuronal survival. Studies have suggested that the release of astroglial and microglial TNF𝛼 may be neuroprotective in a model of ischemic stroke [44,45]. Microglia and astrocytes are also known producers of IL-1α [46], which was also found to be elevated in the brains of offspring in this model and, similar to TNF𝛼, has been suggested to be neuroprotective in models of ischemic stroke [47]. However, whether tissue damage leads to the release of TNFα or IL-1α in a neuroprotective role would need to be confirmed in the context of MAA. Receptors of GM-CSF, another cytokine elevated here in fetal brains after MAA, have been found on microglia, astrocytes, oligodendrocytes, and neurons, with GM-CSF having different dose-dependent effects on each cell type [48].

Ciernia et al. previously reported epigenetic alterations in microglia from adult offspring born to MAA dams. Given the early inflammatory changes observed in the fetal brains in the present study, initial cytokine signals could influence the epigenetic changes to microglia in MAA, as it is known that early life insults to microglia can impact their function [49,50]. Notably, during neurodevelopment, microglia remove unneeded neuronal precursors [51], and when this function is disrupted, increases in neuronal connectivity may result [52]. Expression data from microglia in our model suggest a deviation from normal functioning [23]. Over-connectivity is a phenomenon that has been observed in some cases of ASD and has been linked to changes in social interactions, as well as increased restricted and repetitive behaviors [53,54,55], changes that our model demonstrates [20]. The cascade of inflammatory factors, specifically IL-6, IL-1𝛼, GM-CSF, and TNF𝛼, seen in the fetal brain after MAA highlight an important set of immune markers during fetal development that have neurodevelopmental impacts in shaping the brain architecture and function that may influence the behavioral, neuroinflammatory, and epigenetic alterations observed in adult offspring [20,22,23].

In addition to sex-independent changes in cytokine levels in MAA offspring, we also observed sex-specific changes in placental and fetal brain cytokines. Sex differences are commonly noted in neurodevelopmental disorders [56,57]. For instance, ASD has been shown to occur with a 4:1 ratio for boys to girls [2,3,16]. In the placenta, we observed a decrease in the expression of IL-4, IP-10, and RANTES in male placentas and lower levels of MCP-1 and IL-17 in female placentas. Additionally, the brain cytokine analysis in our model showed a decrease in IL-1β and an increase in MIP-2 in the fetal brains of male offspring taken at GD17.5 but not female offspring. While previous reports have shown an increase in brain IL-1β following MIA induction in both male and female mice [30], the driving mechanisms behind the observed sex differences in our model are yet unclear. The similarities in our findings to clinical reports highlight the significance of our model, and the contrasts to other animal models of maternal immune activation highlight both the novelty of the MAA model and the need for further investigation into mechanistic pathways.

Our fetal brain data reflect an analysis of whole-brain homogenates, an important limitation that may mask more region-specific shifts in brain neuroinflammation. For example, changes in IL-1β in response to maternal poly(I:C) exposure vary by region in the newly born offspring [58], and small elevations in one region—for example, the frontal cortex—can become undetectable when pooled with other regions (e.g., hippocampus). As a result, future studies will require analysis of region-specific changes in fetal brain tissue to get a better understanding of how MAA may be altering brain maturation and differentiation. It is also unclear yet how these changes in the maternal environment communicate changes through the maternal–fetal interface to influence fetal neurodevelopment.

These limitations notwithstanding, our data demonstrate that exposures to allergic asthma inflammation during pregnancy impacts cytokine levels in fetal placenta and brains in a sex-specific manner. Our findings underscore the importance of the maternal environment in shaping fetal brain development and further support behavioral and clinical findings linking MAA to offspring behavioral deficits and neurodevelopmental disorders. Our evidence of early-life programming through maternal immune activation raises important questions surrounding the role of the placenta and immune signals of the brain in shaping offspring neurodevelopment and highlights the importance of understanding the maternal–fetal mechanisms that shape behavioral and mental health later in life.
